# Hemithorax white-out due to massive pleural effusion

**DOI:** 10.1186/s12245-022-00465-x

**Published:** 2023-01-23

**Authors:** Shreya Kolluri, Rohan K. Mangal, Thor S. Stead, Latha Ganti

**Affiliations:** 1 Central Magnet School, Murfreesboro, TN USA; 2 University of Miami Miller School of Medicine, Miami, FL USA; 3 The Warren Alpert Medical School, Brown University, Providence, RI USA; 4 University of Central Florida College of Medicine, Orlando, FL 32827 USA

**Keywords:** Pleural effusion, Recurrent, Thoracentesis, Congestive heart failure, Chronic kidney disease

## Abstract

This is a clinical image submission depicting hemithorax white-out due to massive pleural effusion.

A 79-year-old female was sent from the acute rehab facility where she was recovering from pneumonia due to decreased breath sounds on the left. The patient had mild shortness of breath, but otherwise did not have complaints. Her medical history was significant for dementia, atrial fibrillation, hypertension, chronic kidney disease, and congestive heart failure. Imaging revealed a complete opacification of the left hemithorax consistent with a large pleural effusion (Figs. 1, 2, and 3).

**Fig. 1:**
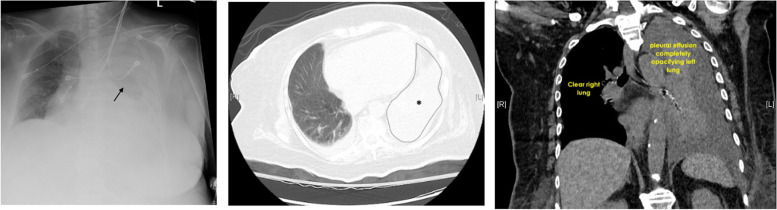
AP chest radiograph, and axial and saggital views of chest CT demonstrating left lung white out

**Fig. 2:**
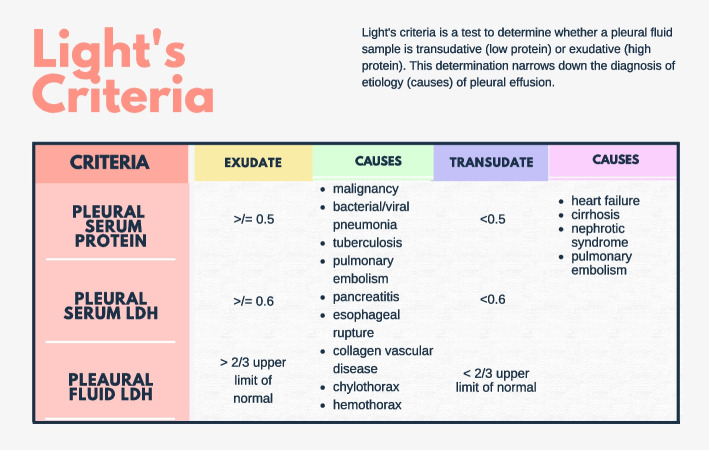
Infographic depicting Light’s Criteria explanation of distinguishing transudate and exudate pleural fluid. Designed by Shreya Kolluri on canva.com

**Fig. 3:**
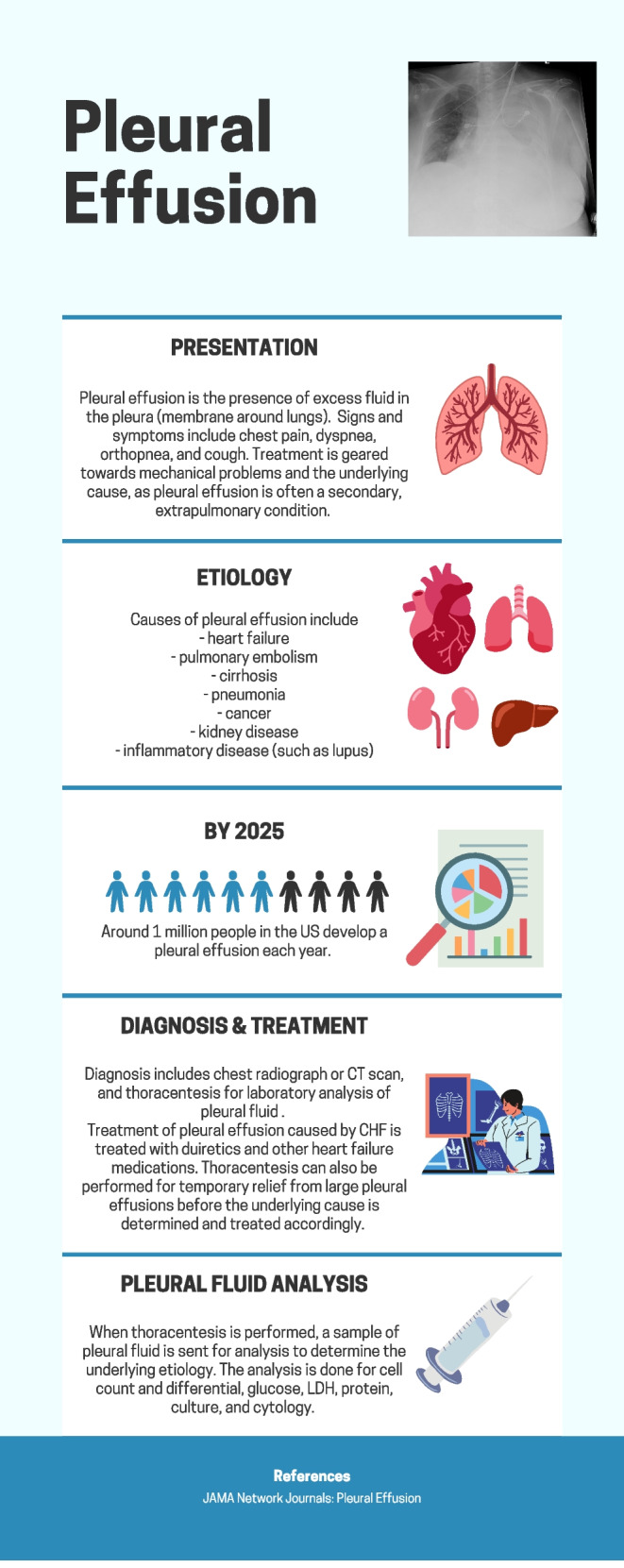
Infographic depicting overview of pleural effusion presentation, symptoms, etiology, and treatment. Designed by Shreya Kolluri on canva.com

About 1.5 million Americans experience pleural effusions annually [1]. Pleural effusion is the accumulation of excess fluid in the membrane around the lungs. The pressure of the fluid on the lungs can result in chest pain, dry cough, dyspnea, and orthopnea, while it can also present with little to no symptoms.

It is often diagnosed with chest radiographs and computed tomography (CT) scans. Chest CT can detect pleural fluid as little as 2 mL as well as underlying abnormalities, such as pneumonia, abscess, or malignant masses [2–4]. Pleural effusion on radiographs appears as opacity because of fluid accumulation between the lower lung and diaphragm [5]. Additionally, thoracic ultrasonography and pleural fluid analysis can be performed to distinguish between transudative and exudative causes as determined by Light’s criteria.

## Data Availability

Data sharing not applicable to this article as no datasets were generated or analyzed during the current study.
